# Large-scale virtual screening on public cloud resources with Apache Spark

**DOI:** 10.1186/s13321-017-0204-4

**Published:** 2017-03-06

**Authors:** Marco Capuccini, Laeeq Ahmed, Wesley Schaal, Erwin Laure, Ola Spjuth

**Affiliations:** 1 Department of Information Technology, Uppsala University, Box 337, 75105 Uppsala, Sweden; 2 Department of Pharmaceutical Biosciences, Uppsala University, Box 591, 75124 Uppsala, Sweden; 3 Department of Computational Science and Technology, Royal Institute of Technology (KTH), Lindstedtsvägen 5, 10044 Stockholm, Sweden

**Keywords:** Virtual screening, Docking, Cloud computing, Apache Spark

## Abstract

**Background:**

Structure-based virtual screening is an in-silico method to screen a target receptor against a virtual molecular library. Applying docking-based screening to large molecular libraries can be computationally expensive, however it constitutes a trivially parallelizable task. Most of the available parallel implementations are based on message passing interface, relying on low failure rate hardware and fast network connection. Google’s MapReduce revolutionized large-scale analysis, enabling the processing of massive datasets on commodity hardware and cloud resources, providing transparent scalability and fault tolerance at the software level. Open source implementations of MapReduce include Apache Hadoop and the more recent Apache Spark.

**Results:**

We developed a method to run existing docking-based screening software on distributed cloud resources, utilizing the MapReduce approach. We benchmarked our method, which is implemented in Apache Spark, docking a publicly available target receptor against $\sim$2.2 M compounds. The performance experiments show a good parallel efficiency (87%) when running in a public cloud environment.

**Conclusion:**

Our method enables parallel Structure-based virtual screening on public cloud resources or commodity computer clusters. The degree of scalability that we achieve allows for trying out our method on relatively small libraries first and then to scale to larger libraries. Our implementation is named Spark-VS and it is freely available as open source from GitHub (https://github.com/mcapuccini/spark-vs).

**Graphical abstract:**
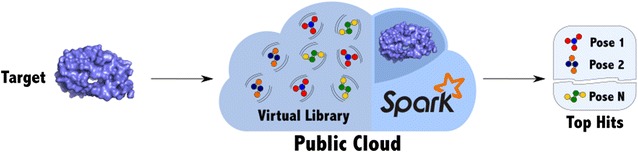
.

## Background

Identification of new drug leads is a key process in drug development. In the pharmaceutical industry, a widely established approach for this is High-Throughput Screening (HTS), where large molecular libraries are screened against a bioassay in fully automated environments [1]. However, HTS is expensive and it has been shown to only produce a small number of hits or to have too many false positives and false negatives [2]. Structure-based virtual screening (SBVS) is a complementary in silico method [3] that has been successfully used to generate new drug leads [4, 5]. SBVS is cheaper and faster than HTS and it can be used in the early stages of drug development to predict if a chemical is likely to interact with certain targets. Typically, a docking-based SBVS workflow starts from a virtual molecular library and a target receptor structure. First, a preliminary preprocessing step, which is subject to the application scope, is performed. We don’t discuss the preprocessing phase in this paper as it depends on several parameters and because publicly-available, ready-to-use libraries exist (e.g., ZINC [6]). After the preprocessing step, molecular docking software is used to dock each chemical in the library to the target receptor. For each molecule in the library, this step will produce a pose (which represents the orientation of the molecule in the target’s pocket) and a score. The higher the score, the more likely the predicted interaction is going to happen in reality. Hence, in the last SBVS phase, all of the poses are sorted by score and the desired number of top hits are returned.

In recent decades, methods in high-throughput structural biology allowed the production of massive virtual molecular libraries. ZINC [6] represents an excellent example, as it contains 20M commercially available molecules in ready-to-dock format. Using large datasets in SBVS is challenging, due to the computational cost. However, SBVS is trivially parallelizable and many tools (e.g., Multilevel Parallel Autodock 4.2 [7] and OEDocking [8]) parallelize it through message passing interface [9] (MPI). Unfortunately, MPI implementations have some disadvantages. In fact, MPI just offers software developers an Application Programming Interface (API) for message passing. This means that, when writing MPI applications, the software developer has to write extra code to manage locality-aware scheduling, load balance and fault tolerance. As a result, many MPI-based applications must rely on fast network connections to provide scalability and in most cases they are not able to complete the computation in the event of a hardware fault. Therefore, to effectively run MPI-based applications, organizations need to have access to High Performance Computing (HPC) facilities.

Google pioneered Big Data analytics on inexpensive hardware with its MapReduce (MR) programming model (and implementation) [10]. In MR applications, problems like data distribution and locality-aware scheduling are managed by the underlying MR implementation, which is transparent to the software developer. In addition, the MR implementation takes care of possible hardware faults, so that the analysis can complete even if some cluster nodes die or if the network becomes temporary unavailable. These key features of MR make MR applications ready to be run in the cloud, where the virtual infrastructure is somewhat comparable to commodity hardware (in terms of performance and reliability), with almost no effort. Even if Google’s MapReduce implementation is not publicly available, some open source implementations exist (Apache Hadoop [11] is probably the most widely used).

Google’s MR has some limitations. MR is based on an acyclic data flow model, which penalizes many popular applications where the same dataset needs to be accessed in multiple iterations (e.g., machine learning and graph algorithms) [12]. In fact, the lack of features like dataset caching, accumulators and broadcast variables, and native workflows support, makes it hard to develop scientific applications. Apache Spark is an open source cluster-computing framework for the processing of large-scale datasets, which overcomes the limitations of MR, while retaining scalability and fault tolerance [12].

Resilient Distributed Datasets (RDDs) represent the core component of Spark [13]. An RDD is an abstraction of a dataset that is partitioned through the cluster and that can therefore be operated on in parallel. RDDs offer almost the same primitives of a standard Scala [14] collection, adding transparent support for locality-aware scheduling, fault tolerance and in-memory dataset caching. Finally, RDDs can be loaded from any mountable distributed file system, Hadoop Distributed File System (HDFS) or any other Hadoop-compatible input source. However, when data is read from a distributed file system, locality-aware scheduling cannot be carried out since such file systems do not provide enough information to the Spark engine.

The applicability of MR-oriented frameworks to virtual screening has already been investigated. For instance, Ahmed et al. [15] implemented ligand-based virtual screening in Spark, showing good scalability in a cloud environment. Zhao et al. [16] described a Hadoop-based infrastructure aimed to make the storage of massive molecular libraries and the docking procedure easier perform for chemists. AutoDockCloud by Ellingson and Baudry [17] is another Hadoop molecular docking implementation. Nevertheless, in the two SBVS studies, the authors show performance metrics running their tools against only a few thousand molecules on bare-metal, high-performance clusters. Furthermore, the Hadoop nature of these projects limits their performance, since Spark-based applications are overall faster (as Shi et al. pointed out [18]).

In this paper we introduce a method for large-scale SBVS on public cloud resources. This represents an advancement over the earlier work by Ellingson and Baudry, as they acknowledged that further refinement in AutoDockCloud was needed in order to run their method on public cloud providers [17]. Furthermore, to the best of our knowledge, in this paper we report for the first time a success story on scaling SBVS over millions of molecules, using public cloud resources. This achievement is relevant for organizations without access to an HPC system, since public cloud resources are readily available with a pay-per-use pricing model, without an upfront cost.

## Results

We developed a method for parallel SBVS following the MR approach, which enables the screening of large molecular libraries on public cloud resources or on commodity hardware clusters. The method is implemented in Apache Spark and it is distributed as an open source library, named Spark-VS, along with some example ready-to-run SBVS pipelines.

**Fig. 1:**
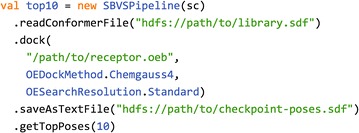
SBVS pipeline in Spark-VS. This example pipeline reads a molecular library in SDF format, docks it against a target receptor and returns the 10 top-scoring molecules. The dock primitive takes as parameters a receptor structure in the OEDocking TK binary format, a scoring method and a search resolution for the underlying docking software. In addition, the *saveAsTextFile* primitive is used to checkpoint all of the poses after the docking phase. This is a best practice as docking is time consuming

Using Spark-VS, the user can define custom SBVS pipelines with a high level API (which is based on the Scala programming language [14]). Despite the fact that the user needs to be familiar with some basic concepts in Spark and Scala, we believe this to be particularly convenient as SBVS applies to many use cases and the user may want to fine-tune the workflows. Figure 1 shows an example pipeline that was defined using Spark-VS. Once the pipeline has being defined, it can be packaged like a standard Spark application to be submitted to a Spark cluster. When using public cloud resources, the effort of starting a Spark cluster boils down to the execution of a setup script.

### Data input

When loading a text file to an RDD, Spark normally splits it line-by-line. Nevertheless, most molecular 3D representations consist of multiple lines. The Structure Data File (SDF) is a handy, multiline format that can be used to store 3D molecular structures along with some metadata (e.g., identifier and docking score) [19]. Spark-VS can read SDF files and properly split them across the cluster thanks to a custom record reader that we defined. Since each instance of the docking program takes some time to initialize, it is good to feed each docking process with multiple molecules. On the other hand, if too many are passed together, load balancing becomes harder. For this reason, the custom SDF input format takes a *chunk size* parameter (default: 30) that controls the number of molecules to be loaded into a single RDD record.

### Parallel screening

OEDocking TK [20] was used as the underlying docking software. Even if this is a commercial software, free academic licenses are available. OEDocking TK provides a C++ API that we used to implement a light-weight docking executable, which takes a chunk of molecules from the standard input, docks them to a particular receptor (available on each node through the *addFile* primitive) and produces a chunk of poses (with the relative score) in the standard output. Using the standard input and the standard output in this way is very convenient. In fact, Spark-VS uses the *map* RDD primitive to pipe each RDD record to a docking executable instance via standard input and takes the results back via standard output. This contrasts to the implementations by Zhao et al. [16] and by Ellingson and Baudry [17] where the input chunks are passed to the docking program and read back to the Hadoop framework using local files. This impacts performance, since unlike pipes (that are fully operated in memory), files are stored to the disk.

RDDs offer some convenient primitives to do the post processing part of SBVS. For instance, Spark-VS uses the *saveAsTextFile* primitive to handle persistence on a distributed storage. However, the built-in *sortBy* primitive is not suitable for SBVS. The Spark primitive assumes small RDD records so shuffling the relatively large SDF representations through the network would lead to a large overhead. Fortunately, we figured out a simple workaround to avoid this. Instead of performing distributed sorting, Spark-VS collects ID/Score tuples for each pose and efficiently sorts them serially. Then, using the RDD *filter* primitive, Spark-VS retrieves the top scoring molecules by ID.

With the aim of testing our parallel implementation, we ran OEDocking TK serially over 1000 molecules that were randomly drawn from the benchmark dataset. We observed that the output for the parallel implementation did not differ from the one that we got in the serial execution. We have provided this validation procedure along with Spark-VS and it can be easily reproduced by running in local mode.

### Experiments

#### Experimental settings

We deployed a standalone Spark cluster, along with HDFS, on the CityCloud [21] public cloud provider using SparkNow [22] for host cloud and virtual machine provisioning. More specifically, we setup 21 nodes with 4 virtual CPUs (vCPUs), 8 GB of RAM, 20 GB of ephemeral storage and 40 GB of block storage each. This is a fully virtualized environment that, in terms of resources, resembles a commodity computers cluster. Since in a Spark cluster, one node (namely, the master node) acts as a controller, the maximum level of parallelism was 80 in our case.

#### Benchmark

We benchmarked our method and the Spark-VS implementation, screening HIV-1 protease receptor against the whole SureChEMBL library [23] downloaded in ready-to-dock format from ZINC. The raw SureChEMBL dataset contains 17M compounds, retrieved from patent documentation. However, the ready-to-dock version contains only $\sim$2.2 M molecules, as ZINC applies some filtering rules in its preparation protocol. The dataset, in SDF format, was made available to the worker nodes via HDFS (using block size 64 Mb and block redundance 3). It is interesting to observe that this dataset is relatively small in terms of disk space ($\sim$8 GB), if compared to more orthodox MR benchmarks. In fact, Google claims to use MR for the processing of petabytes of data [10]. Nevertheless, molecular docking is compute intensive, which contrasts to traditional MR applications and it justifies the applicability of Spark to such use case.

Finally, it is worth mentioning that the target receptor was converted in the OEDocking TK format starting from an HIV-1 protease receptor representation available in the literature [24].

#### Performance metrics

**Fig. 2:**
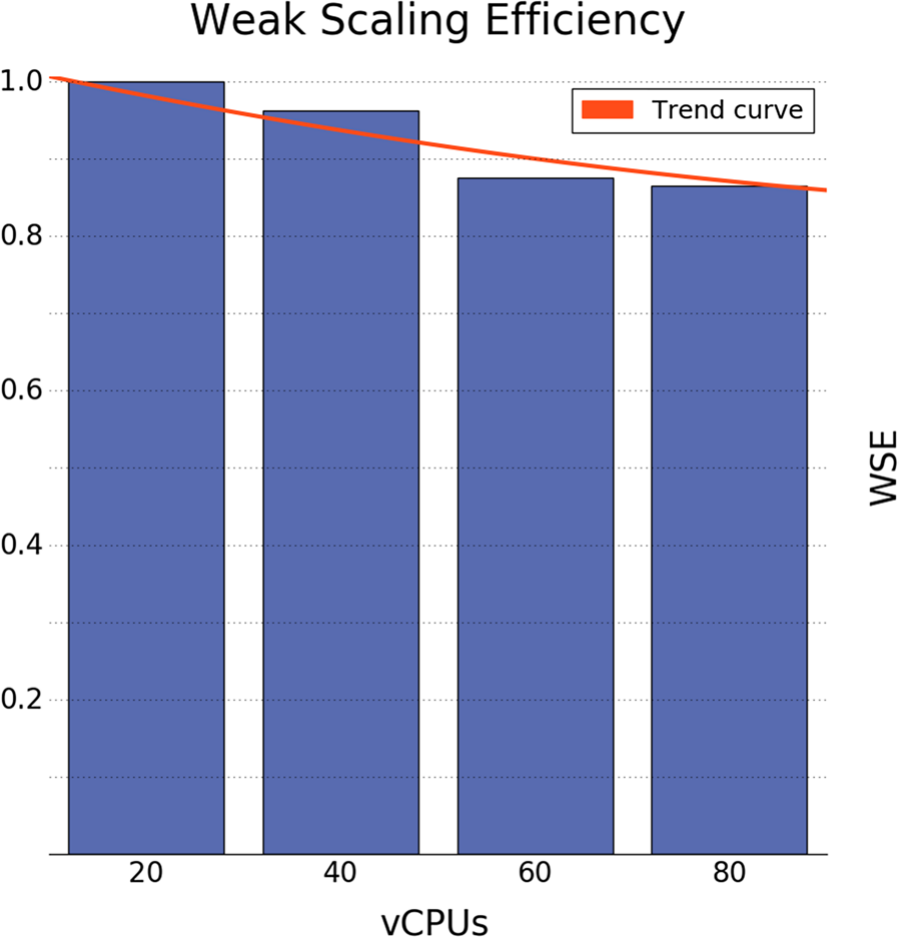
Weak Scaling Efficiency plot. Each bar represents a different run and it shows how efficiently the respective vCPUs were used. The input size was increased by a work unit, along with the number of vCPUs, in each consecutive run. The trend curve was computed by 2nd degree polynomial interpolation

First, we studied the scaling efficiency of Spark-VS, pointing out the portion of processing units that are actually used during the computation. The scaling efficiency is a very important matter when using cloud resources since they are typically pay-per-use. In order to give a resource usage estimation, we repeatedly ran Spark-VS over $1/4, 2/4 \ldots 4/4$ of the benchmark dataset, restricting the vCPUs usage to 20 in the first run, and allowing 20 additional vCPUs each time the input increased. Then, for each run we computed the Weak Scaling Efficiency (WSE), that is the running time for one processing element (20 cores in our case) to process one work unit (1/4 of the dataset), divided by the running time for *N* processing elements to process *N* work units ($N=1,2 \ldots 4$ in our case). Figure 2 shows the WSE for each run. An important finding is that, when using full resources, we are able to show a scaling efficiency of 87%.

**Fig. 3:**
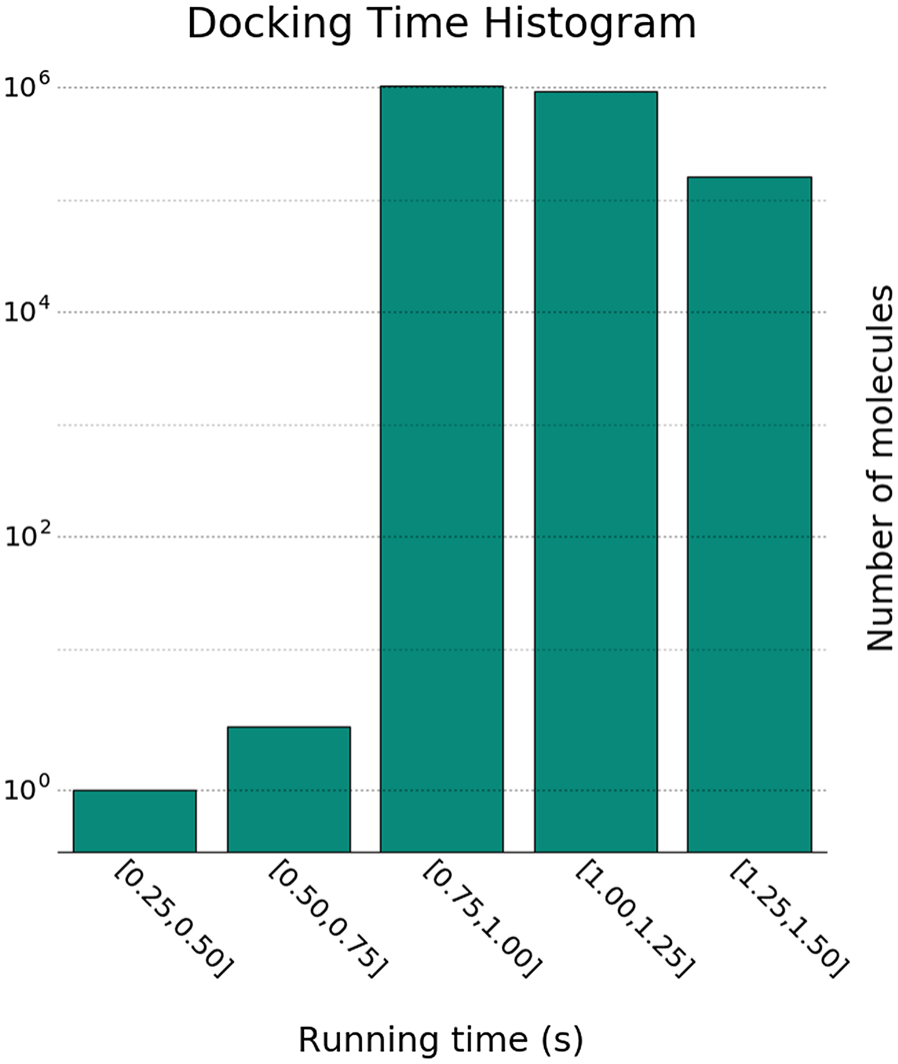
Docking time per molecule. The histogram shows the serial docking time for each molecule in the benchmark dataset (${\sim}2.2\,M$) divided into equally spaced bins. Note that in this plot the *number of molecules* is on logarithmic scale

It took 8.9 h to run the complete analysis on 80 vCPUs. The speedup is an interesting metric that compares the parallel running time, to the single core running time. Given the single core running time $T_1$, and the parallel running time $T_N$, where *N* is the parallelism level, the speedup is defined as $T_1/T_N$. In other words, the speedup tells us how much faster the computation get completed, using a certain level of parallelism. In order to compute *T*1 when running the analysis, we annotated each pose with the time that it took to produce it on the assigned vCPU. The histogram in Fig. 3 shows the 2.2 M running times, in equally spaced bins. The serial running time, for each of the molecules in the benchmark, sums up to $\sim$635.7 h. Hence, we got a speedup of $\sim$71 when using 80 cores.

## Discussion

From the experiments, it emerged that our method scales well. In fact, we got a scaling efficiency of 87% and a speedup of 71, when using 80 vCPUs. These two metrics are strictly related, since a good speedup can be obtained only when the resources are efficiently exploited. The benchmark experiments showed that the WSE levels off when increasing from 60 to 80 vCPUs, and the running time allows for running the analysis over night. Hence, we believe that the costs for more massive runs are not justified.

Molecular docking is compute intensive, which contrasts to most of the MR applications, where a quick operation is applied to each input record. In Fig. 3, we observe that the docking time varies between 0.75 and 1.50 for most of the molecules. This is an important remark, as Spark does not have enough information to predict how long the processing of each record will take, hence it assigns molecules to processing units randomly. Therefore, we believe that the scaling efficiency of the method can be improved by tuning the Spark cluster configuration, for better load balancing. We leave this as future work, since it is not the focus of this study.

Ellingson and Baudry reported a speedup of 450 for AutoDockCloud, when they ran it on a high-performance bare-metal Hadoop installation [17]. Comparing the speedup that we got for Spark-VS is unfair, as we ran in a fully virtualized environment with a much lower parallelism level. Nowadays, the biggest cloud providers (e.g., Amazon Web Services [25] and Google Cloud Platform [26]) sell their resources as virtual infrastructure, providing handy tools to deploy Spark and Hadoop clusters. This is why we believe that MR applications should always be tested on virtual environments. Fortunately, Ellingson and Baudry provide enough information to estimate their scaling efficiency. AutoDockCloud was tested on 2637 input molecules, with a parallelism level of 570. Therefore, we can derive a work unit of ${\sim}5$ molecules. The total serial running time for 2637 molecules was ${\sim}69$ h. Hence, each molecule was docked in 94 s on average, leading to a average work unit time of 470 s. The parallel execution of AutoDockCloud took 550 s, therefore we can estimate a WSE of 85%, which is slightly lower than the one we show for Spark-VS (87%).

## Conclusion

Our method provides the means to run SBVS on public cloud resources or on commodity computers clusters. The performance experiments showed good scalability on a virtual environment that resembles an inexpensive hardware cluster. To the best of our knowledge, this is the first time that a method has been shown to scale SBVS over millions of molecules on a fully virtualized environment. This is a very important point, because public cloud providers, such as Amazon or Google, only sell virtual resources. Public cloud is pay-per-use and it can be allocated and deallocated on-demand. Furthermore, cloud providers offer handy tools and interfaces that provide the means to setup Hadoop and Spark clusters, relieving the scientists from tedious configurations. Hence, being able to scale SBVS in these kinds of environments constitutes an important advancement. In fact, organizations that want to approach SBVS can now try it out on their favorite cloud provider, benchmarking it on relatively small libraries (at lower costs), and then scale to larger libraries. Furthermore, being relieved form the up-front investments, hardware configuration and maintenance costs is certainly an advantage too.

The method, along with its source code and unit tests, is free to use and publicly available on GitHub [27]. Even though our implementation uses a commercial molecular docking software by default, which is free only for academics, any other docking software can be used with minor adaption.

## References

[1] Fox S, Farr-Jones S, Sopchak L, Boggs A, Nicely HW, Khoury R, Biros M (2006). High-throughput screening: update on practices and success. J Biomol Screen.

[2] Hughes JP, Rees S, Kalindjian SB, Philpott KL (2011). Principles of early drug discovery. Br J Pharmacol.

[3] Cheng T, Li Q, Zhou Z, Wang Y, Bryant SH (2012). Structure-based virtual screening for drug discovery: a problem-centric review. AAPS J.

[4] Seifert MH, Lang M (2008). Essential factors for successful virtual screening. Mini Rev Med Chem.

[5] Villoutreix BO, Eudes R, Miteva MA (2009). Structure-based virtual ligand screening: recent success stories. Comb Chem High Throughput Screen.

[6] Irwin JJ, Sterling T, Mysinger MM, Bolstad ES, Coleman RG (2012). ZINC: a free tool to discover chemistry for biology. J Chem Inf Model.

[7] Norgan AP, Coffman PK, Kocher JP, Katzmann DJ, Sosa CP (2011). Multilevel parallelization of Auto Dock 4.2. J Cheminform.

[8] OEDocking. http://www.eyesopen.com/oedocking-v3.2-released. Accessed 13 July 2016

[9] Forum MPI (1994). MPI: a message-passing interface standard. Int J Supercomput Appl.

[10] Dean J, Ghemawat S (2008). MapReduce: simplified data processing on large clusters. Commun ACM.

[11] Apache Hadoop. https://hadoop.apache.org. Accessed 13 July 2016

[12] Zaharia M, Chowdhury M, Franklin MJ, Shenker S, Stoica I (2010) Spark: cluster computing with working sets. In: Proceedings of the 2Nd USENIX conference on hot topics in cloud computing., HotCloud’10USENIX Association, Berkeley, CA, USA, pp 10–10

[13] Zaharia M, Chowdhury M, Das T, Dave A, Ma J, McCauley M, Franklin MJ, Shenker S, Stoica I (2012) Resilient distributed datasets: a fault-tolerant abstraction for in-memory cluster computing. In: Proceedings of the 9th USENIX conference on networked systems design and implementation., NSDI’12USENIX Association, Berkeley, CA, USA, pp 2–2

[14] Scala. http://scala-lang.org. Accessed 13 July 2016

[15] Ahmed L, Edlund Å, Laure E, Spjuth O (2013) Using iterative MapReduce for parallel virtual screening. In: IEEE 5th international conference on cloud computing technology and science, CloudCom 2013, Bristol, United Kingdom, vol 2, pp 27–32

[16] Zhao J, Zhang R, Zhao Z, Chen D, Hou L (2012) Hadoop mapreduce framework to implement molecular docking of large-scale virtual screening. In: Proceedings of the 2012 IEEE Asia-Pacific services computing conference)., APSCC ’12IEEE Computer Society, Washington, DC, USA, pp 350–353

[17] Ellingson SR, Baudry J (2011) High-throughput virtual molecular docking: Hadoop implementation of autodock4 on a private cloud. In: Proceedings of the second international workshop on emerging computational methods for the life sciences., ECMLS ’11ACM, New York, NY, USA, pp 33–38

[18] Shi J, Qiu Y, Minhas UF, Jiao L, Wang C, Reinwald B, Özcan F (2015). Clash of the titans: mapreduce vs. spark for large scale data analytics. Proc. VLDB Endow..

[19] Dalby A, Nourse JG, Hounshell WD, Gushurst AKI, Grier DL, Leland BA, Laufer J (1992). Description of several chemical structure file formats used by computer programs developed at molecular design limited. J Chem Inf Comput Sci.

[20] OEDocking TK. http://www.eyesopen.com/oedocking-tk. Accessed 13 July 2016

[21] City Cloud. https://www.citycloud.com. Accessed 13 July 2016

[22] SparkNow. https://github.com/mcapuccini/SparkNow. Accessed 13 July 2016

[23] Papadatos G, Davies M, Dedman N, Chambers J, Gaulton A, Siddle J, Koks R, Irvine SA, Pettersson J, Goncharoff N, Hersey A, Overington JP (2016). SureChEMBL: a large-scale, chemically annotated patent document database. Nucleic Acids Res..

[24] Bäckbro K, Löwgren S, Österlund K, Atepo J, Unge T, Hultén J, Bonham NM, Schaal W, Karlén A, Hallberg A (1997). Unexpected binding mode of a cyclic sulfamide HIV-1 protease inhibitor. J Med Chem.

[25] Amazon Web Services. https://aws.amazon.com. Accessed 13 July 2016

[26] Google Cloud Platform. https://cloud.google.com. Accessed 13 July 2016

[27] Spark-VS GitHub Page. https://github.com/mcapuccini/spark-vs. Accessed 13 July 2016

